# Honey bee foraging ecology: Season but not landscape diversity shapes the amount and diversity of collected pollen

**DOI:** 10.1371/journal.pone.0183716

**Published:** 2017-08-30

**Authors:** Nadja Danner, Alexander Keller, Stephan Härtel, Ingolf Steffan-Dewenter

**Affiliations:** Biocenter, University of Würzburg, Würzburg, Germany; University of Cologne, GERMANY

## Abstract

The availability of pollen in agricultural landscapes is essential for the successful growth and reproduction of honey bee colonies (*Apis mellifera* L.). The quantity and diversity of collected pollen can influence the growth and health of honey bee colonies, but little is known about the influence of landscape structure on pollen diet. In a field experiment, we rotated 16 honey bee colonies across 16 agricultural landscapes, used traps to collect samples of collected pollen and observed intra-colonial dance communication to gain information about foraging distances. DNA metabarcoding was applied to analyze mixed pollen samples. Neither the amount of collected pollen nor pollen diversity was related to landscape diversity. However, we found a strong seasonal variation in the amount and diversity of collected pollen in all sites independent of landscape diversity. The observed increase in foraging distances with decreasing landscape diversity suggests that honey bees compensated for lower landscape diversity by increasing their pollen foraging range in order to maintain pollen amount and diversity. Our results underscore the importance of a diverse pollen diet for honey bee colonies. Agri-environmental schemes aiming to support pollinators should focus on possible spatial and temporal gaps in pollen availability and diversity in agricultural landscapes.

## Introduction

Pollen is the main protein source for honey bees and also provides lipids, vitamins and minerals [1]. The availability of pollen is essential for the growth, development and reproduction of honey bee colonies. A honey bee colony in temperate zones collects about 15–30 kg of pollen per year [2, 3], almost all of which is consumed, with a reserve of about 1 kg kept in the colony at any time [4, 5]. Colony survival is positively influenced by the pollen amount collected in the course of the season [6]. Therefore, pollen resources must be available continuously to assure sufficient pollen supply. Besides quantity [6], the diversity of collected pollen is important for honey bee health [7, 8]. Honey bee colonies forage on an area of more than 10 km^2^ and several studies have reported on the diversity of collected pollen [2, 9–12]. But the ways in which land use change and agricultural intensification influence the amount and diversity of collected pollen remains largely unexplored. Earlier studies have indicated that foraging distances of honey bee colonies increase in structurally simple landscapes with a high proportion of arable land and depend on the availability of crop-based pollen resources [13, 14]. Honey bees expanded their foraging range for pollen when there was less semi-natural habitat (SNH) with a diversity of resources available [15]. Landscape composition (an indicator of landscape structure) can be characterized not only by the area of specific habitat types such as SNH but also by landscape diversity, which evaluates richness and evenness aspects of the landscape, i.e. the number and area of different habitat types [16, 17]. We assume that landscape diversity (based on the Shannon index) provides a good indication of the potential diversity of available resources. It may influence foraging distances as well as the amount and diversity of the pollen diet, which has rarely been addressed [18]. Less diverse landscapes with lower resource availability—except in the case of mass-flowering crops—and diversity may lead to a lower input and diversity of pollen. However, since honey bees are able to discriminate between different pollen diets [19] and rely on a wide variety of resources [11, 20] they might compensate for lower landscape diversity—in cases where resources are insufficient—by increasing their foraging range. Pollen availability is a function not only of landscape diversity but is also subject to seasonal fluctuations. For example, the presence of certain mass-flowering resources is mostly influenced by season. Oilseed rape OSR flowers in April and May while other mass-flowering crops, such as sunflower and maize, reach their peak later in summer. Colonies also have seasonal shifts in their pollen demands. Together these shifts in flowering resources and colony demands could change the types of pollen collected over time.

A major restriction to research on the foraging ecology of honey bees is the time-consuming identification of pollen grains via light microscopy and its limited taxonomic resolution, typically to the family level [21]. Next-generation sequencing presents a useful and efficient approach to identify pollen at the genus and species levels without the need for specialised palynological expert knowledge [21]. Sequencing data can also be used for abundance estimation. Subsequent analysis of species composition and diversity (Shannon index) provides insight into the pollen resource use of pollinators and its variability. In this study we analyzed pollen foraging of honey bee colonies in differently structured agricultural landscapes across the season to answer the following questions: (1) Does the amount of pollen collected (dry weight) depend on landscape diversity or season? (2) Is the richness and diversity of pollen influenced by landscape diversity or season? (3) Do honey bees compensate for a lower landscape diversity by increasing their pollen foraging range to maintain pollen amount and diversity? (4) Which are the most abundant pollen taxa and how do they vary over time?

## Materials and methods

### Study region and experimental design

**Fig 1:**
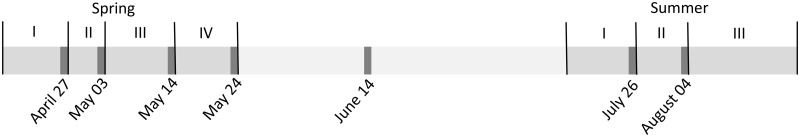
Timeline of sampling. Timeline of the study period from 18/04/12 to 20/08/12. Seven days of pollen sampling in 16 landscapes are shown in dark grey, seven periods of waggle dance observation (observation rounds) in middle grey and no data collection in light grey. Spring I—Summer III refer to the seven observation rounds as described in subsection Experimental design.

This study was conducted around Würzburg, Germany, where the landscape is dominated by agriculture which includes cultivation of mass-flowering crops such as oilseed rape. Semi-natural habitats (SNH) comprise flower rich calcareous grasslands, extensive meadows and hedges. They are present to a varying extent. We used 16 observation hives, each with a honey bee colony of approximately 4000 worker bees on two brood frames, for observation of waggle dances and collection of pollen samples. We selected 16 landscapes with 2 km radius each and with an overall gradient in landscape diversity. Each observation hive was set up in the center of one landscape (see Table 1 for geographic coordinates). All hives were located on private land with the permission of the respective land owners. Seven observation rounds were performed from 18 April to 20 August 2012 (Fig 1). Each observation round included a 90-minute observation period for each colony (observing all 16 colonies took several days per observation round), a full day of collecting pollen loads simultaneously from all colonies, and a final rotation of all hives to be set up in another landscape for the next observation round (except for the last round). The rotation of the hives followed a fixed scheme that was logistically determined and that assured a minimum 10 km relocation distance of each colony, which prevented them from flying back to the previous landscape. The rotation allowed for data collection from seven independent colonies in each landscape. This landscape scale experiment was—except for the pollen collection—described in a previous study by the authors [15]. Waggle dance data were re-analyzed in the context of landscape diversity. Pollen data were described and analyzed for the first time within the present study.

**Table 1:** Working names of bee hive locations and the respective Gauß-Krüger coordinates.

Location	X	Y
Biebelried	4361525.04	5514464.00
Birkenfeld	4335245.56	5528080.69
Duttenbrunn	4335527.60	5532119.83
Erlabrunn	4344482.75	5525759.62
Estenfeld	4358389.81	5522563.62
Euerfeld	4363414.90	5523594.12
Gambach	4340210.49	5542669.64
Gerbrunn	4356469.11	5517113.54
Greussenheim	4338443.76	5522383.23
Karsbach	4342204.00	5548109.00
Marktbreit	4364603.26	5504662.36
Obersfeld	4348993.00	5546000.00
Pleichfeld	4361351.67	5528186.77
Retzstadt	4347758.00	5531357.00
Waldbrunn	4342262.54	5518361.09
Winterhausen	4355570.37	5508294.96

### Observation and decoding of waggle dances

Waggle dances of bees returning with pollen loads were observed to calculate foraging distances for pollen as described in [15]. The duration of a series of circuits and the corresponding number of circuits were recorded for each bee carrying pollen and dancing at least five consecutive circuits [22]. Foraging distance (*y*) was calculated via the mean duration of a single dance circuit (*x*) according to a polynomial fit (*y* = 92.137 − 346.659 * *x* + 228.454 * *x*^2^ − 10.963 * *x*^3^) based on data by [23]. Only dances of bees carrying pollen and with five consecutive circuits were decoded [22].

### Landscape diversity

We measured landscape diversity via the Shannon index, which is widely used and recommended for landscape management in an ecological framework [17]. It is defined as

$$\begin{array}{c} SHDI=-\sum_{i=1}^{N}P_{i}\cdot \ln P_{i} \end{array}$$

where N is the number of habitat types and *P*_*i*_ is the proportional abundance of habitat type i. Landscape diversity is independent of specific habitat types and therefore suitable for comparison between studies. The calculation of landscape diversity for all 16 landscapes was based on the area of seven main land use types within 2 km (Table 2; [14, 15]). Vector data for cropland, grassland, other agricultural land, hedges, forest and settlement was obtained from Bayerisches Landesvermessungsamt. Further, vector data for semi-natural habitat was obtained from the Bavarian mapping of biotopes (Bayerisches Landsamt für Umwelt).

**Table 2:** Land-use characteristics for 16 landscapes in a 2000 m radius around observation hives. Means ± standard errors and ranges (%) are shown.

Habitat type	Mean ± SE	Range
Crop total	66.2 ± 4.0	37.8–93.3
Forest	16.7 ± 3.0	0.1–33.6
Settlement	7.1 ± 0.8	2.3–13.8
Seminatural habitat	4.6 ± 1.2	0.2–14.3
Other agricultural land	3.2 ± 1.3	0.0–18.1
Grassland	4 ± 0.7	0.3–10.6
Hedges	2.1 ± 0.7	0.0–9.5

### Pollen sampling and analysis

We used self-built pollen traps that were designed to fit on the entrances of observation hives and recently used by [24]. They are in principle similar to pollen traps for full sized hives. Returning foragers had to pass a 5mm hole grid and pollen was collected in a drawer underneath. Pollen sampling at all colonies was performed on six dates within the observation rounds and one additional date in June. Pollen loads were collected at night from the drawers and frozen at −20°C. For further analysis pollen samples were dried at 40°C and 30% relative humidity for 48 hours. The dry weight of all samples was determined. Samples were homogenized before taking sub-samples. DNA from about 0.003 *g* pollen grains was isolated as described by [21] using the Macherey-Nagel Food Kit (Düren, Germany) and the supplementary protocol of the kit dedicated to pollen samples. The amplification PCR for the ITS2 marker was performed in three separate 10 *μL* reactions in order to avoid PCR bias [25]. Primers were ITS-S2F [26] and ITS4R [27], but modified for sample multiplexing according to [28].

Each reaction contained 5 *μL* 2x Phusion Master Mix (New England Biolabs, Ipswich, MA, USA), 0.33 *μM* each of the forward and reverse primers, 3.34 *μL* PCR grade water and 1 *μL* DNA template. PCR conditions were as follows: initial denaturation at 95°C for 4 *min*, 37 cycles of denaturation at 95°C for 40 *s*, annealing at 49°C for 40 *s* and elongation at 72°C for 40 *s*; followed by a final extension step at 72°C for 5 *min*. Triplicate reactions of each sample were combined after PCR and DNA amounts between samples were normalized using the SequalPrep Normalization Plate Kit (Invitrogen GmbH, Darmstadt, Germany). Pooled multiplexed samples were quality controlled using a Bioanalyzer High Sensitivity DNA Chip (Agilent Technologies, Santa Clara, CA, USA) and quantified with the dsDNA High Sensitivity Assay (Life Technologies GmbH, Darmstadt, Germany). Sequencing was performed on the Illumina MiSeq using 2x150 cycles v2 chemistry (Illumina Inc., San Diego, CA, USA). Raw sequence data was deposited at the European Nucleotide Archive (ENA, http://www.ebi.ac.uk/ena) with the project accession number PRJEB15870.

Raw reads were joined using QIIME v1.8.0 [29] and filtered with USEARCH v8.0.1477 [30] to remove low quality data (< *Q*20, < 150 *bp*, ambiguous base-pairs). Reads were classified first by a global query with USEARCH with a threshold of > 97% sequence identity. For this, only reference sequences of plants occurring in Bavaria according to http://www.bayernflora.de (accessed on 2015/01/24) were extracted as a subset from the complete ITS2-database [31], covering 90.4% of genera known in this area. Sequences that did not match such references were then classified to the highest possible taxonomic group, but maximally genus, using UTAX as implemented in USEARCH v8.0.1477 [30], the UTAX-reference database of [28] and the scripts for parsing deposited in https://www.github.com/iimog/meta-barcoding-dual-indexing [28].

Data were imported into R [32] using the phyloseq package [33]. We filtered Chlorophyta from the dataset and transformed raw read numbers into relative amounts. Species below a minimum relative abundance of 1% per sample were removed. Six of 102 samples were omitted due to an insufficient number of reads, determined via rarefaction curves of all samples. We calculated the richness per sample as the number of different plant species from which the pollen orginated. As a measure of diversity, we calculated the Shannon index on a per sample basis by applying it to the number of sequencing reads per plant taxon. Here, sequencing reads are the estimate for abundance of the respective plant taxon. Pollen samples were comparable between landscapes and seasonal dates due to the standardized colony sizes throughout the experiment.

### Statistical analysis

We analyzed dry weight, richness and diversity of pollen samples, each in response to season (date), landscape diversity and their interaction using the open source statistical software R [32]. Response variables were square root transformed if necessary. Linear mixed models were applied using the package nlme [34] for the analysis of dry weight and richness. Landscape was included as random factor. We checked for temporal autocorrelation within the models. A generalized least squares model with a weights function (to account for variance heterogeneity between dates) and a correlation structure (to account for replication of sites) was applied to analyze pollen diversity. A linear mixed model was applied using the package lme4 [35] to analyze log-transformed pollen foraging distances. Site and colony were implemented as crossed random factors, date and landscape diversity as interacting explanatory variables. The relative importance of explanatory variables was evaluated for each model based on model selection via AICc comparison with the function “dredge” (multi-model inference; applied to the full model) and model averaging [36] over the full set of possible models (R package MuMIn; [37]). It was calculated for each variable by the sum of weights across all models within a set in which the respective variable occurred and can take values between 0–1 [36]. The values are given in the text and results of “dredge” in the supplement (Tables A-D in S1 File). Only important variables and interactions (importance > 0.5) were included in the final model. On the final models post hoc comparisons after Tukey with “BH” corrections were applied [38] and results are shown within the figures. In all cases model diagnostic plots were checked for validity of the full model.

## Results

### Dry weight of pollen samples

**Fig 2:**
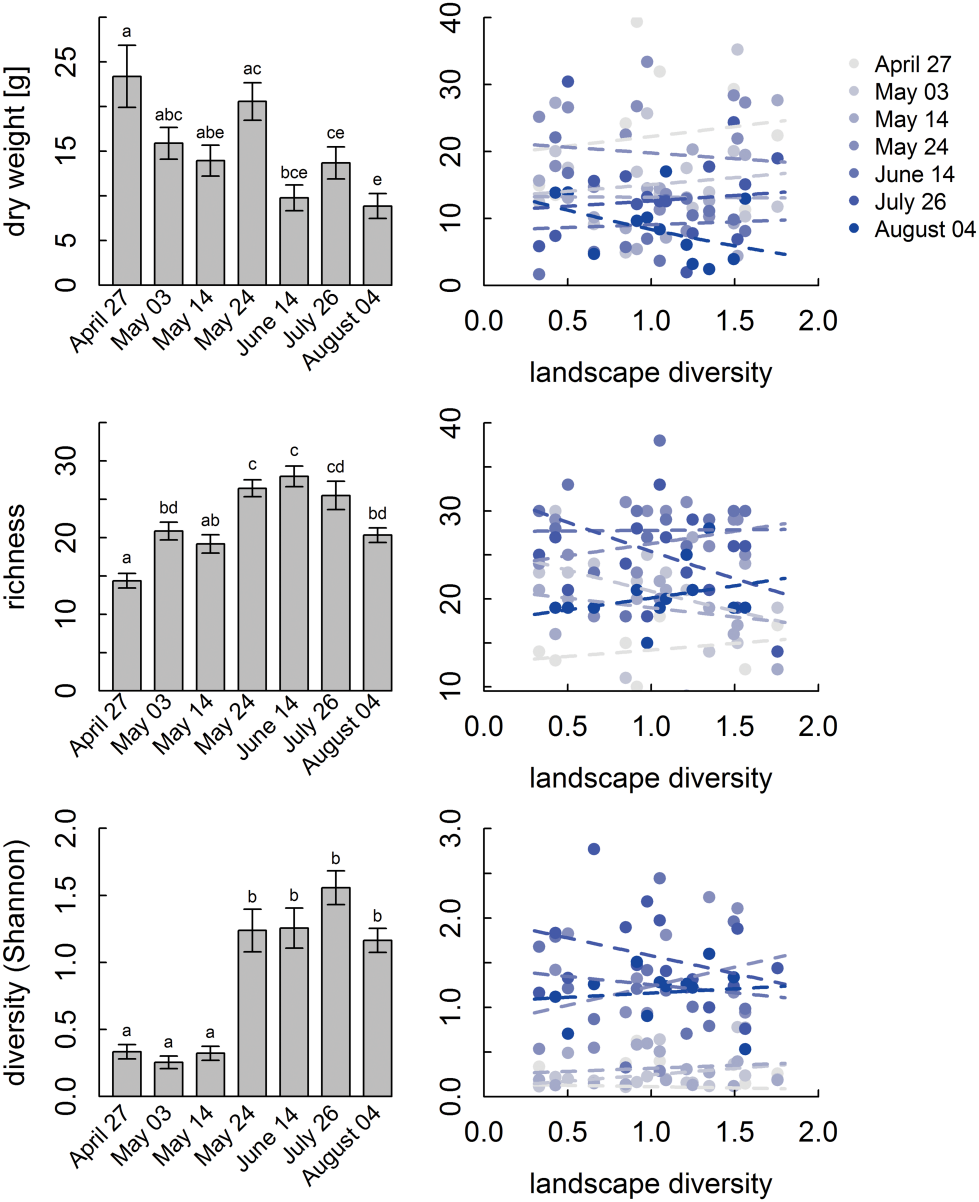
Dry weight, richness and diversity of pollen samples. Different letters above bars indicate significant differences (*P* < 0.05). The respective relationships to landscape diversity in interaction with date are shown on the right and are not significant. Pollen was collected by honey bee colonies on seven dates in 16 landscapes.

Dry weight of pollen samples ranged from 1.7 to 39.3 g per day with a mean of 14.7 g (±0.8 *s*.*e*.*m*.) over the whole study period. In contrast to expectations, the amount of pollen collected per day did not depend on landscape diversity (importance: date 1, landscape diversity 0.23, interaction < 0.01; Fig 2; Table 3; Table A in S1 File). However, we observed a significant seasonal variation in the amount of pollen collected with highest values in April and May (for results of post hoc test see Fig 2). We also checked to determine whether seasonal variation in the amount of collected pollen varied more in simple compared to diverse landscapes, but the interaction between landscape and season was not important.

**Table 3:** Results of model selection analyzing dry weight, richness and diversity of pollen samples collected by honey bee colonies. The 95% best models (sum of weight 0.95) per response variable are shown. Explanatory variables are D = date, L = landscape diversity, and R = observation round. Weight is the AIC weight compared to all possible models. See Fig 1 for the difference between date and observation round.

Response	Model specification	*R*^2^	df	AICc	Δ AIC	weight
Dry weight	D	0.30	9	269.4	0.00	0.78
	D + L	0.30	10	271.9	2.48	0.22
Richness	D	0.43	9	203.1	0.00	0.62
	D + L	0.44	10	204.1	0.99	0.37
Diversity	D	0.77	15	86.7	0.00	0.78
	D + L	0.77	16	89.0	1.96	0.22
Distance	R + L + R:L	0.30	17	3222.8	0.00	0.97

### Richness and diversity of pollen samples

**Fig 3:**
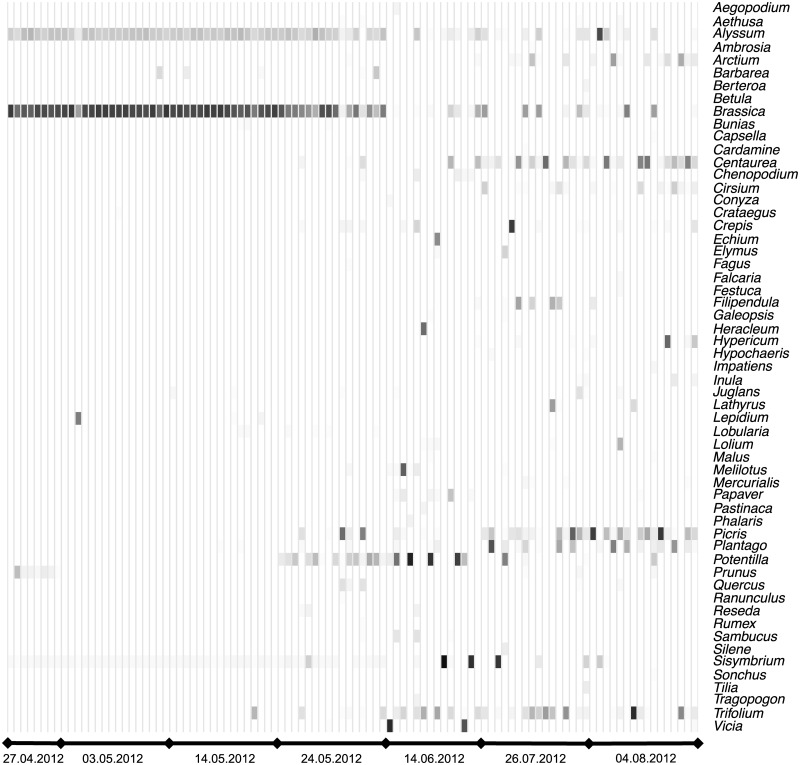
Heatmap of genera composition of pollen samples at seven dates. Each column presents an individual sample. Each row is a genus that was recorded with relative abundance values above the detection threshold at least within one sample. Darkness intensity of the cells indicate relative abundance of sequence reads within the sample, with white = 0% and black = 100%.

DNA sequencing of the mixed pollen samples permitted us to determine the plant species from which pollen originated. In total, we generated 1529901 quality filtered sequencing reads, with an average throughput of 15936 reads per sample (± SD 11775). We detected 80 taxa from 56 genera with an abundance of DNA reads >1% per sample (*N* = 102; Fig 3; S1 Table). Seventy-five taxa could be determined to species level and five to genus level. The species richness of pollen samples ranged from 9 to 47 species per sample with a mean of 23 (± 0.6 s.e.m.). Pollen richness did not depend on landscape diversity and its interaction with date (Fig 2; Table B in S1 File). Again, season had a strong effect on pollen richness (importance: date 1, landscape diversity 0.38, interaction < 0.01; Table 3) with significantly lower values in April and the first half of May compared to the ends of May, June and July. Similarly, pollen diversity (Shannon index) was independent of landscape diversity but varied significantly by date (importance: date 1, landscape diversity 0.22, interacion < 0.01; Table C in S1 File). It ranged from 0.11–2.77 and was significantly lower in April and the first half of May compared to all other dates.

### Pollen foraging range

**Fig 4:**
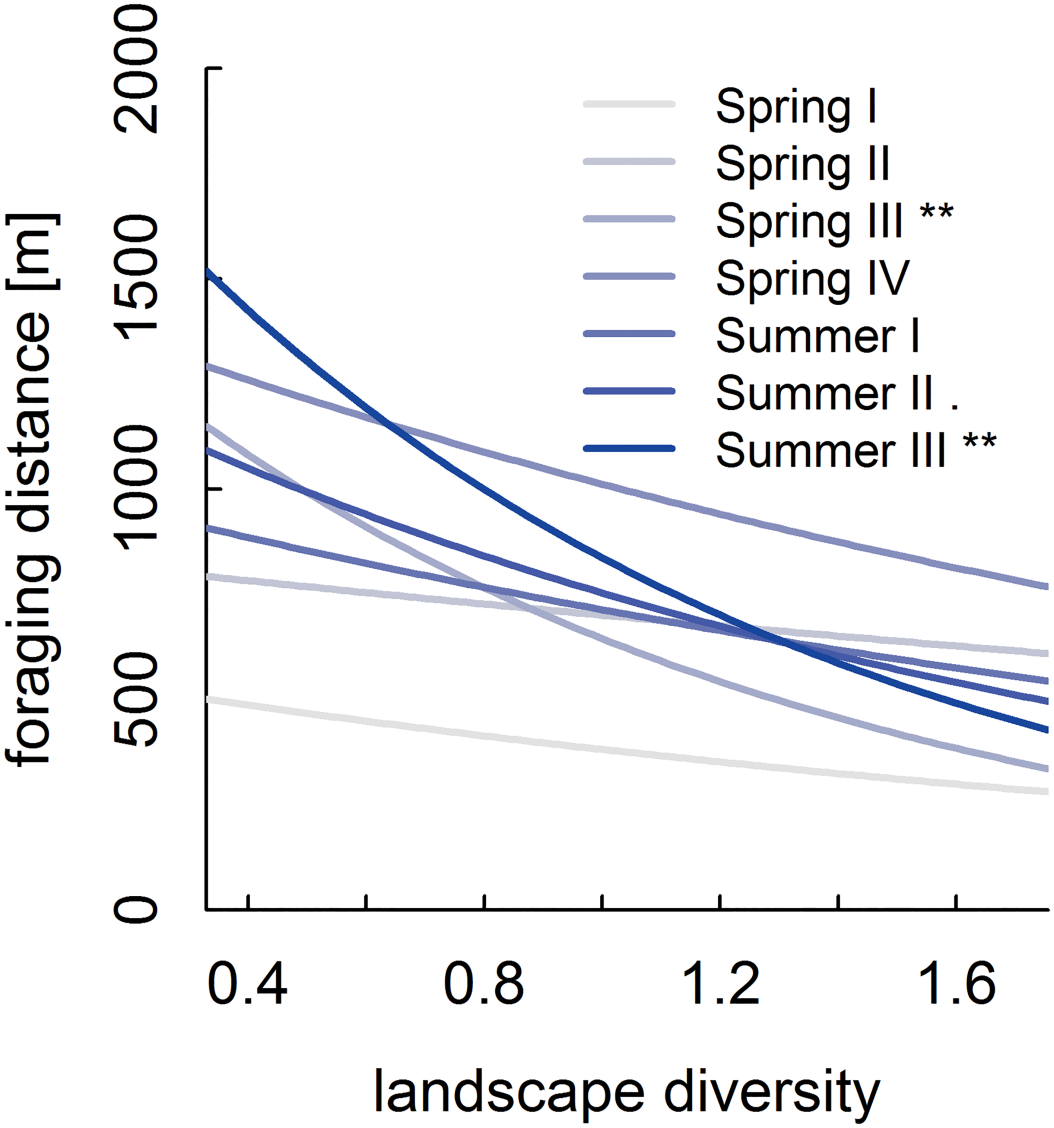
Pollen foraging distances of honey bees in relation to landscape diversity. Data were collected based on observation of waggle dances in eight observation rounds in 16 landscapes. Slopes significantly different from zero: · *P* < 0.1; ** *P* < 0.01. For the distribution of sampling dates, see Fig 1.

We hypothesized that honey bee colonies would increase their foraging range in landscapes with low habitat and thus floral resource diversity in order to maintain a constant amount and diversity of incoming pollen. Indeed, foraging distances for pollen significantly increased with decreasing landscape diversity, depending on date (importance: date 1, landscape diversity 1, interaction 0.97; for results of post hoc test see Fig 4; S1 Fig and Table D in S1 File).

### Dominant taxa

The relative abundance of sequencing reads within a sample was used as an approximation of estimated pollen quantity from a specific taxon. Overall, pollen from *Brassica napus* (48%) was most abundant followed by *Papaver rhoeas* (6%), *Picris hieracioides* (6%), *Centaurea jacea* (5%) with all other species < 5% abundance. At the genus level, *Brassica* (49%) was followed by *Papaver* (6%), *Picris* (6%), *Trifolium* (5%) and *Centaurea* (5%). On family level *Brassicaceae* (55%) were followed by *Asteraceae* (16%), *Fabaceae* (7%), *Rosaceae* (7%) and *Papaveraceae* (6%). *Brassica napus* was dominant in April and May (86% ± 0.06 s.e.m.). In addition to *Brassica napus*, *Prunus avium* was present in a low proportion in April, and *Potentilla spec*. likewise at the end of May. In June, July and August there appeared to be no similarly dominant genera. *Papaver rhoeas* was the most abundant species in June (35%), *Picris hieracioides* in July (21%) and *Centaurea jacea* in August (25%).

## Discussion

DNA metabarcoding of mixed pollen samples collected by honey bee colonies revealed interesting insights into honey bee foraging in agricultural landscapes. We could not find any relationship between dry weight, richness and diversity of pollen samples with landscape diversity and its interaction with date. However, in all cases, we detected a dependency on season (date). Importantly, our results indicate that honey bees compensated for less diverse landscapes by increasing their foraging range in order to maintain the amount and diversity of collected pollen.

In order to compare the amount (dry weight) of pollen collected in our study with data from other studies colony size must be taken into account. The average of 15 g pollen per day for colonies with an average size of 4000 workers in our study would approximately correspond to daily 75 g for colonies of 20000 workers if simply multiplied. Such colonies have a daily pollen intake of approximately 30 g (Danner et al., unpublished) and the collected amount of pollen in this study seems to be rather high by comparison. However, the efficiency of pollen traps might vary [39] and data are not always comparable between studies. The observed seasonal differences in dry weight with the highest values in April and May may in part reflect colony development, with higher protein demands for larvae rearing in spring. In contrast to [11], we observed a peak of collected pollen in April, which coincides with the mass-flowering of oilseed rape. However, the very low input in June coincides with the food depletion period reported by [11]. Occurring when colonies are at their maximum population size, it could affect colony growth rates and health [11].

Collected pollen came from a wide variety of plant species (80). Our result lies in between previous studies of 46 [9] and 95 for feral honey bee colonies [10], but much below the values of 164 and 228 reported by [12] and [11] for managed honey bee colonies (*Apis mellifera carnica*). However, if our dataset was expanded to include species with a minimum relative abundance of 0.1% per sample instead of 1%, pollen from 149 different plant species was detected. The additional species did not change the patterns of richness and diversity but increase their levels almost equally over all samples. We excluded taxa below 1% abundance, however, because their contribution was low in the overall composition of pollen. We also wanted to reduce the risk of including cross-contamination by wind and other pollinators or of introducing sequencing errors into the analysis in our study.

Richness of pollen was lower in April and first half of May compared to end of May and June and this pattern was even stronger with respect to pollen diversity. Lower values in spring corresponded to the mass-flowering of oilseed rape and the higher demand of colonies for pollen. They collected the bulk of their pollen diet from oilseed rape to meet the demand during a stage of naturally strong colony growth. In contrast, previous studies have reported a low probability of collecting pollen from oilseed rape [11, 40, 41]. In a previous analysis within the same field experiment [15] we assessed the role of oilseed rape by calculating foraging frequencies for different land use types based on decoded waggle dances. We found that during the flowering period of OSR, recruiting foragers refer significantly more frequently to SNHs as compared to mass-flowering OSR fields. In contrast, our DNA metabarcoding data of pollen collections from the same colonies indicated a preference for OSR pollen. However, this apparent discrepancy between the decoded behavioural pollen foraging pattern and the metabarcoding data of the collected pollen samples is based on relative preferences for different habitat types. Per area SNH, honey bee colonies dance twice as often for rewarding resources than for OSR. However, a larger percent area of OSR fields compared to SNH could still result in a significant proportion of OSR pollen collected by colonies in April and May. Nonetheless, our study suggests that in comparison to OSR crop fields, honey bee colonies preferentially communicate diverse pollen resources located in habitats with patchy plant distribution. Interestingly, this could be an adaptation to ensure the collection of diverse pollen sources with known benefits for bee health. Further, analyses of metabarcoding sequence data, which require a PCR amplification step, have shown some limitations in terms of quantification [28]. The proportion of OSR pollen could be over-amplified in the PCR leading to an over-representation of the related sequence reads. We accounted for this effect by preparing triplicate samples following a study that directly compared pollen abundances by light-microscopy and metabarcoding, but the effect may not have been completely removed [21].

Our study supports the hypothesis that honey bees try to collect a diverse pollen diet in order to ensure colony performance [7, 11, 15, 19]. First, during mass-flowering of oilseed rape in April and May honey bees still foraged for supplementary pollen from about 20 plant species (±2.49 s.e.m.; each > 1% abundance) per landscape, and this constituted about 4–25% of the daily input, depending on date. This richness seems high in comparison with results from Israel, where the number of pollen sources from trapped pollen pellets ranged between 5 and 20 plant species per sampling date per site [2]. A further indication of a pollen diversity driven foraging strategy is provided in a recent study by [24], who showed that bee communication appears to be necessary especially for pollen foraging. They observed higher communication efforts for pollen resources located within habitats with higher plant biodiversity. However, the higher effort may also have been related to a higher resource patchiness of SNHs. A recent laboratory experiment demonstrated that honey bees are able to balance colony nutrition deficiencies by selectivity for different pollen [19], while our data provides evidence for it under field conditions. Similarly, [11] reported unexpectedly high pollen diversity during oilseed rape bloom. Second, landscape diversity (and therefore diversity of resources) did not influence the amount and diversity of collected pollen, as could be assumed if honey bees had been foraging randomly. [18] also report that the diversity of pollen, collected by honey bees in a field experiment, does not reflect landscape diversity. Finally, in the Northern Great Plains region of the US, the diversity of bee-collected pollen was not correlated to large-scale land use patterns [42].

We demonstrated that foraging distances increase with decreasing landscape diversity. At the same time, neither pollen amount nor diversity were influenced by landscape diversity, suggesting that honey bees compensate for lower resource availability by increasing their foraging range to maintain pollen amount and diversity. Similarly, [13] reported higher pollen foraging distances in simple landscapes compared to complex ones which presumable offer more resources, and [15] found that pollen foraging distances increased when semi-natural habitats close to the hive decreased. Results of [15] are based on the same landscape scale experiment as the recent study. In this study we focussed on pollen diversity and on a more general measure of landscape composition—landscape diversity instead of SNH area—which allows for a more general interpretation and comparison with other (future) studies. The diversity of pollen input, as shown in our study, is most probably a result of nutritional demands that must be met; they apparently can be met regardless of landscape diversity, but at the costs of higher foraging distances. In other words, the effectiveness of a colony decreases in less diverse landscapes, as more effort has to be put into foraging for pollen. If we consider landscapes with even lower diversity (as is the case in many agricultural regions within Germany, but also worldwide) than in our study, a honey bee colony could come to a point where the costs exceed the benefits, i.e. when it is no longer worthwhile to expand the foraging range to increase pollen diet diversity. However, foraging distances up to 13.5 km [23] have been reported. The importance of a diverse pollen diet for honey bee health is confirmed by [7] who found that it can enhance immune functions of honey bees, and that, conversely, minimal nutrient diversity may not meet all honey bee needs. In support of this, positive effects of pollen diet diversity on the lifespan of parasitized honey bees have been reported [8].

### Conclusions

In conclusion, the amount and diversity of pollen input were influenced by season but not by landscape diversity, suggesting that, at least in the case of small honey bee colonies, demands can be met in both simple and complex agricultural landscapes. In contrast to results from previous studies, oilseed rape was an important pollen resource in the current study, resulting in lower pollen diversity in spring. However, gaps in availability that can not be compensated by further extending the foraging range might arise if alternative resources outside mass-flowering periods are missing. Agri-environmental schemes aiming to support pollinators should develop adequate floral resources for agricultural landscapes which fill the temporal and spatial gaps in resource availability and diversity. The successful application of DNA metabarcoding in our study underscores the utility of this novel approach to understanding honey bee foraging ecology that also reduces reliance on specific palynological expert knowledge.

## Supporting information

S1 Fig Pollen foraging distances versus landscape diversity. Raw data points for seven observation rounds in 16 landscapes (8 landscapes in the first round).
(TIFF)

S1 Table List of species. (PDF)

S2 Table Original data of foraging distances. (ODS)

S3 Table Original data of pollen samples. (ODS)

S1 File Results from multi-model inference. (PDF)
