# Supplementary material for: Honey bee foraging ecology: Season but not landscape diversity shapes the amount and diversity of collected pollen
Source: PLoS One. 2017 Aug 30;12(8):e0183716. doi: 10.1371/journal.pone.0183716 (PMC5576699; doi:10.1371/journal.pone.0183716)
Supplement: S1 Table — (PDF) [file pone.0183716.s002.pdf]

Table S1: List of species, sorted by relative abundance over all samples. All species had an abundance of >1% within the samples in which they occurred.

**Genus**	**Species**	**Abundance [%]**
Brassica	*napus*	48.38
Papaver	*rhoeas*	6.43
Picris	*hieracioides*	6.16
Centaurea	*jacea*	4.91
Alyssum	*murale*	2.65
Potentilla	*micrantha*	2.04
Trifolium	*pratense*	1.96
Cirsium	*palustre*	1.67
Trifolium	*repens*	1.56
Vicia	*lathyroides*	1.49
Sisymbrium	*irio*	1.26
Filipendula	*ulmaria*	1.22
Crepis	*vesicaria*	1.07
Trifolium	*hybridum*	0.94
Potentilla	*reptans*	0.8
Hypericum	*perforatum*	0.71
Trifolium	*striatum*	0.63
Plantago	*major*	0.62
Heracleum	*mantegazzianum*	0.61
Lepidium	*perfoliatum*	0.6
Prunus	*avium*	0.57
Sisymbrium	*officinale*	0.56
Malus	*domestica*	0.55
Potentilla	*clusiana*	0.51
Arctium	*tomentosum*	0.49
Brassica	*nigra*	0.4
Barbarea	*vulgaris*	0.34
Melilotus	*officinalis*	0.3
Plantago	*lanceolata*	0.28
Carthamus	spec.	0.26
Potentilla	*alba*	0.25
Sisymbrium	*austriacum*	0.22
Juglans	*regia*	0.22
Centaurea	*cyanus*	0.21
Mercurialis	*annua*	0.18
Chenopodium	*ficifolium*	0.18
Inula	*germanica*	0.17
Potentilla	*norvegica*	0.17
Neslia	*paniculata*	0.15
Quercus	*robur*	0.13
Reseda	*lutea*	0.11
Potentilla	*caulescens*	0.11
Tilia	*platyphyllos*	0.08
Lolium	*multiflorum*	0.07
Hypochaeris	*radicata*	0.07
Crataegus	*monogyna*	0.06
Elymus	*repens*	0.06
Trifolium	*arvense*	0.06
Silene	*vulgaris*	0.05
Tragopogon	*pratensis*	0.05

Sisymbrium	*loeselii*	0.05
Sisymbrium	*strictissimum*	0.04
Berteroa	*incana*	0.04
Mercurialis	spec.	0.04
Phalaris	*arundinacea*	0.04
Senecio	*doronicum*	0.04
Elymus	*caninus*	0.04
Quercus	*petraea*	0.03
Prunus	*domestica*	0.03
Lathyrus	*pratensis*	0.03
Aethusa	*cynapium*	0.03
Conyza	*canadensis*	0.02
Rosa	*rugosa*	0.02
Ambrosia	*artemisiifolia*	0.02
Fagus	*sylvatica*	0.02
Cardamine	*amara*	0.01
Betula	*nana*	0.01
Crataegus	*laevigata*	0.01
Solanum	*nigrum*	0.01
Ranunculus	*circinatus*	0.01
Jacobaea	spec.	0.01
Capsella	*bursa-pastoris*	0
Sonchus	*oleraceus*	0
Falcaria	*vulgaris*	0
Daucus	*carota*	0
Impatiens	*glandulifera*	0
Lolium	*perenne*	0
Hypericum	*hirsutum*	0
Quercus	spec.	0
Onobrychis	spec.	0
