# Supplementary material for: Honey bee foraging ecology: Season but not landscape diversity shapes the amount and diversity of collected pollen
Source: PLoS One. 2017 Aug 30;12(8):e0183716. doi: 10.1371/journal.pone.0183716 (PMC5576699; doi:10.1371/journal.pone.0183716)
Supplement: S1 File — (PDF) [file pone.0183716.s005.pdf]

Table A: Dry weight - results from multi-model inference.

Component models:

	R^2	df	logLik	AICc	delta	weight
1	0.30	9	-124.65	269.39	0.00	0.78
12	0.30	10	-124.64	271.87	2.48	0.22
123	0.32	16	-123.45	285.78	16.40	0.00
(Null)	0.00	3	-141.94	290.14	20.75	0.00
2	0.00	4	-141.93	292.30	22.91	0.00

Term codes:

date	landscape_diversity	date:landscape_diversity
1	2	3

Table B: Richness - results from multi-model inference.

Component models:

	R^2	df	logLik	AICc	delta	weight
1	0.43	9	-69.65	159.39	0.00	0.62
12	0.44	10	-68.93	160.44	1.05	0.37
123	0.49	16	-64.67	168.22	8.84	0.01
(Null)	0.00	3	-96.89	200.03	40.65	0.00
2	0.02	4	-96.11	200.67	41.28	0.00

Term codes:

date	landscape_diversity	date:landscape_diversity
1	2	3

Table C: Diversity - results from multi-model inference.

Component models:

	R^2	df	logLik	AICc	delta	weight
1	0.77	15	-25.24	86.49	0.00	0.78
12	0.77	16	-25.05	88.98	2.49	0.22
123	0.78	22	-22.34	102.55	16.06	0.00
(Null)	0.49	9	-62.75	145.59	59.10	0.00
2	0.49	10	-62.74	148.07	61.58	0.00

Term codes:

date	landscape_diversity	date:landscape_diversity
1	2	3

Table D: Foraging distances - results from multi-model inference.

Component models:

	R^2	df	logLik	AICc	delta	weight
123	0.30	17	-1594.17	3222.79	0.00	0.97
12	0.30	11	-1603.98	3230.16	7.37	0.02
2	0.28	10	-1606.89	3233.95	11.15	0.00
1	0.23	5	-1655.62	3321.28	98.49	0.00
(Null)	0.23	4	-1657.77	3323.57	100.77	0.00

Term codes:

landscape_diversity	round	landscape_diversity:round
1	2	3
